# Integration of two herbivore‐induced plant volatiles results in synergistic effects on plant defence and resistance

**DOI:** 10.1111/pce.13443

**Published:** 2018-10-16

**Authors:** Lingfei Hu, Meng Ye, Matthias Erb

**Affiliations:** ^1^ Institute of Plant Sciences University of Bern Bern Switzerland

**Keywords:** (*Z*)‐3‐hexenyl acetate, benzoxazinoids, indole, induced resistance, insects, jasmonic acid, maize, plant defence, plant herbivore interactions, volatile communication

## Abstract

Plants can use induced volatiles to detect herbivore‐ and pathogen‐attacked neighbors and prime their defenses. Several individual volatile priming cues have been identified, but whether plants are able to integrate multiple cues from stress‐related volatile blends remains poorly understood. Here, we investigated how maize plants respond to two herbivore‐induced volatile priming cues with complementary information content, the green leaf volatile (*Z*)‐3‐hexenyl acetate (HAC) and the aromatic volatile indole. In the absence of herbivory, HAC directly induced defence gene expression, whereas indole had no effect. Upon induction by simulated herbivory, both volatiles increased jasmonate signalling, defence gene expression, and defensive secondary metabolite production and increased plant resistance. Plant resistance to caterpillars was more strongly induced in dual volatile‐exposed plants than plants exposed to single volatiles.. Induced defence levels in dual volatile‐exposed plants were significantly higher than predicted from the added effects of the individual volatiles, with the exception of induced plant volatile production, which showed no increase upon dual‐exposure relative to single exposure. Thus, plants can integrate different volatile cues into strong and specific responses that promote herbivore defence induction and resistance. Integrating multiple volatiles may be beneficial, as volatile blends are more reliable indicators of future stress than single cues.

## Abstract 2

Plants respond to relatively unreliable individual herbivore‐induced plant volatiles by priming their defenses. Whether they can integrate multiple volatiles as more reliable indicators of herbivory remains unknown. We show that exposure of maize plants to two different volatile cues with complementary information content, (*Z*)‐3‐hexenyl acetate and indole, primes defence responses in a synergistic manner and enhances resistance to caterpillars more strongly than exposure to individual cues. Thus, plants can integrate different volatiles into enhanced defence responses.

## 1 INTRODUCTION

The capacity to perceive and respond to fluctuating environments is essential to all life on earth. As primary producers in terrestrial ecosystems, plants are constantly dealing with limiting resources, adverse abiotic conditions, competitors, pests, and pathogens (Cramer, Urano, Delrot, Pezzotti, & Shinozaki, 2011; Van Dam, 2009). By consequence, they have evolved systems to detect these stressors and respond to them appropriately (Cui, Tsuda, & Parker, 2015; Felton & Tumlinson, 2008; Hirayama & Shinozaki, 2010). Plants can, for example, perceive pathogens and herbivores directly via associated molecular patterns or indirectly via volatile cues from attacked neighbors (Bonaventure, 2012; Heil, 2014; Zipfel, 2014). The induction and priming of defence responses by herbivore‐ and pathogen‐induced volatiles in particular is increasingly recognized as an important aspect of plant immunity and resistance (Baldwin, Halitschke, Paschold, von Dahl, & Preston, 2006; Frost et al. 2008; Heil, 2014; Karban, Yang, & Edwards, 2014; Mescher & De Moraes, 2014; Riedlmeier et al., 2017; Turlings & Erb, 2018).

Although plant perception of individual environmental cues is relatively well understood, less is known about the capacity of plants to integrate multiple environmental cues (Finch‐Savage & Leubner‐Metzger, 2006). Integrating multiple cues may enable plants to obtain more reliable information of a given environmental condition than individual cues. Many volatiles that are released from leaves upon herbivore attack are also released constitutively by other sources, including flowers, bacteria, and fungi (Piechulla, Lemfack, & Kai, 2017; Tholl, Sohrabi, Huh, & Lee, 2011), and thus do not provide reliable information about the presence of an herbivore on a neighbouring plant (Baldwin et al., 2006). By contrast, the overall composition of herbivore‐induced volatile blends is often highly species and stress‐specific and may thus indicate the presence of herbivores more reliably (Junker et al., 2017; McCormick, Unsicker, & Gershenzon, 2012). Whether plants can integrate multiple volatile cues into defence responses is not well understood (Erb, 2018; Ruther & Kleier, 2005).

The perception of herbivore‐induced plant volatiles has been studied in detail in maize (Zea mays). Maize plants that are exposed to volatile blends from herbivore‐attacked plants respond more rapidly and more strongly to subsequent herbivore attack (Engelberth, Alborn, Schmelz, & Tumlinson, 2004; Ton et al., 2007). This form of priming includes higher amounts of jasmonates, higher expression of defence‐related genes, and higher emission of terpene volatiles (Engelberth et al., 2004, Ton et al., 2007). Furthermore, caterpillar growth is reduced and herbivore natural enemies are more strongly attracted to herbivore‐attacked maize plants that are exposed to herbivore‐induced volatiles (Ton et al., 2007). So far, two components of the herbivore‐induced volatile blend of maize have been identified to trigger defence priming. Green leaf volatiles (GLVs) such as (*Z*)‐3‐hexenal, (*Z*)‐3‐hexen‐1‐ol, and (*Z*)‐3‐hexenyl acetate (HAC) can induce and prime the expression of jasmonate biosynthesis genes, the production of jasmonates, and the emission of volatile terpenes (Engelberth et al., 2004). HAC can also modulate defense and growth in other plants such as poplar, lima bean and pepper (Frost et al., 2008, Freundlich & Frost, 2018).The volatile phytohormone ethylene has been shown to increase the release of maize volatiles that are induced by (*Z*)‐3‐hexen‐1‐ol (Ruther & Kleier, 2005). The aromatic volatile indole primes jasmonates and volatile terpenes and is required for within‐plant priming of monoterpenes (Erb et al., 2015). GLVs are specific for plants, but are released in response to many stresses including drought, mechanical wounding, herbivore attack, and pathogen infection (Ebel, Mattheis, & Buchanan, 1995; Scala, Allmann, Mirabella, Haring, & Schuurink, 2013). By contrast, indole is produced by many different organisms and plant tissues (Bailly et al., 2014; Stamm, Lottspeich, & Plaga, 2005), but its release from plant leaves seems to be specific to herbivore attack, as herbivore‐derived elicitors, but not wounding alone induce strong indole emissions (Frey et al., 2000), and the indole biosynthesis gene *ZmIGL* is induced by herbivore attack, but not by other stresses such as salt stress or fungal infection (Erb et al., 2009). Thus, GLVs and indole complement each other in terms of the information they convey, and the simultaneous presence of GLVs and indole may be a better predictor of the presence of a herbivore‐attacked plant than each cue alone. As both GLVs and indole prime jasmonate defenses, it is conceivable that they may have additive effects on defence priming.

Based on these considerations, we investigated how simultaneous exposure of maize plants to HAC and indole affects maize defenses. We first quantified the impact of HAC and indole individually on phytohormone production, defence gene expression, and defence metabolite accumulation in plants that were induced by simulated herbivory and measured the influence of these volatiles on plant resistance to herbivores. We then compared the effects of individual volatile exposure with the effects of simultaneous exposure to HAC and indole. We tested for synergistic effects of HAC and indole exposure by comparing the effects elicited by simultaneous exposure with the calculated additive effects of the individual exposures (Machado, Arce, McClure, Baldwin, & Erb, 2018). Our experiments reveal that maize plants integrate two different herbivore‐induced volatiles into strong and specific defence signatures.

## 2 MATERIALS AND METHODS

### 2.1 Plants and herbivores

The maize (Z. mays) genotype B73 was used in this study. Maize seedlings were grown as previously described (Erb et al., 2011). Fourteen‐day‐old plants were used for all experiments. *Spodoptera littoralis* eggs were provided by the University of Neuchâtel and reared on artificial diet as previously described (Maag et al., 2014). Herbivore oral secretions were collected from third instar S. littoralis larvae, which had been feeding on maize leaves for 48 hr. Briefly, the S. littoralis larvae were held with a pair of lightweight forceps, and regurgitation was induced by gently pinching their heads with another pair of forceps. Oral secretions were collected using a micropipette and collected in Eppendorf tubes on ice. Oral secretions were stored at −80°C and diluted 1:1 in autoclaved Milli‐Q water prior to use.

### 2.2 Volatile dispensers

Volatile dispensers were manufactured as previously described (Erb et al., 2015; von Merey et al., 2011). Dispensers consisted of 2‐ml amber glass vials (11.6 × 32 mm^−2^; Sigma, St. Louis, USA) containing 20 mg of synthetic indole (>98%, GC, Sigma, St. Louis, USA) or 0.2‐ml (*Z*)‐3‐hexenyl acetate (HAC, >98%, Sigma, St. Louis, USA). The vials were closed with open screw caps that contained a PTFE/rubber septum, which was pierced with a 2‐μl micropette (Drummond, Millan SA, Switzerland). The vials were sealed with parafilm and wrapped in aluminium foil for heat protection and to avoid photodegradation. The dispensers release approximately 150 ng h^−1^ of indole and 70 ng h^−1^ of HAC, which corresponds to amounts typically emitted by herbivore‐attacked maize plants (Erb et al., 2015, von Merey et al., 2011). Control dispensers were prepared the same way using empty glass vials. Dispensers were prepared 24 hr before the start of experiments.

### 2.3 Plant volatile exposure

To expose maize plants to synthetic indole and/or HAC, different sets of dispensers were individually introduced into 2‐L glass vessels containing maize seedlings. The glass vessels were connected to a multiple air‐delivery system via PTFE tubing. Purified air entered the glass vessels at a flow rate 0.3 L min^−1^ and was released through additional openings. This set‐up ensured sufficient ventilation to avoid the buildup of unnatural volatile concentrations while effectively isolating the headspaces of the different plants. The volatile exposure system was placed into a greenhouse cabin (26 ± 2°C; 14: 10 hr, light [8 a.m.–10 p.m.]: dark; 55% relative humidity). Dispensers were added into the glass vessels in the evening (8 p.m.) before herbivore induction. The following treatment combinations were used in all experiments: Control (empty dispenser), HAC (HAC dispenser), indole (indole dispenser); HAC + indole (HAC dispenser and indole dispenser). Although HAC is released 1 hr earlier than indole upon simulated herbivory (Erb et al., 2015), both volatiles are released continuously and simultaneously from maize leaves that are attacked by real caterpillars (Erb et al., 2011). We therefore exposed maize plants to HAC and indole using the same timing. After 16 hr of exposure (at 10 a.m.), the plants were carefully removed from the glass vessels, placed on a table in the same greenhouse cabin, and induced as described in the next section.

### 2.4 Plant induction by simulated herbivory

To test how indole and HAC influence herbivore‐induced plant responses, the pre‐exposed maize plants were induced by wounding two leaves over an area of ~0.5 cm^−2^ on both sides of the central vein with a razor blade, followed by the application of 10 μl of S. littoralis oral secretions. This treatment results in plant defence responses similar to real S. littoralis attack (Erb et al., 2009) and is referred to as “simulated herbivory” or “induction” throughout the rest of the manuscript. In three different experiments, leaves were either harvested at 0 min (no herbivore induction), 45 min, or 5 hr after simulated herbivory and then flash frozen and used to quantify phytohormones, expression of defence‐related genes, benzoxazinoids, and volatiles. Whole maize leaves, excluding the damaged area, were harvested. All analyses within time points were performed on the same leaf samples.

### 2.5 Gene expression analysis

The influence of volatile exposure on the herbivore‐induced expression of signalling and defence genes was determined by quantitative real‐time PCR (QRT‐PCR, *n* = 5). On the basis of earlier studies, we measured the induction of hormone biosynthesis genes and hormonal signalling markers 45 min upon simulated herbivory (*n* = 5) and the induction of defence‐related genes 5 hr upon simulated herbivory (*n* = 5; Seidl‐Adams et al., 2015). In addition, we measured the effect of HAC and indole on all marker genes at the 0‐min time point to evaluate direct induction. Maize leaves were ground to a fine powder under liquid nitrogen. Total RNA of 80‐mg maize leaf powder was isolated using the GeneJET Plant RNA Purification Kit (Thermo Scientific, Waltham, MA, USA). Three hundred nanograms of total RNA of each sample were then reverse transcribed with the SuperScript® II Reverse Transcriptase (Invitrogen, Carlsbad, CA, USA). The QRT‐PCR assay was performed on the LightCycler® 96 Instrument (Roche, Switzerland) using the KAPA SYBR FAST qPCR Master Mix (Kapa Biosystems, Wilmington, MA, USA). The maize actin gene *ZmActin* was used as an internal standard to normalize cDNA concentrations (Erb et al., 2009). The relative gene expression levels of the target genes were calculated using the 2^−ΔΔCt^ method (Wong & Medrano, 2005). The primers of all tested genes are provided in Table S1.

### 2.6 Phytohormone analysis

The influence of volatile exposure on herbivore‐induced phytohormone levels were measured 45 min after induction by simulated herbivory (*n* = 5). This time point was selected on the basis of established hormone accumulation kinetics and volatile priming effects, both of which peak at 35–45 min after herbivore induction in maize (Engelberth et al., 2004; Erb et al., 2015). Jasmonic acid (JA), 12‐oxophytodienoic acid (OPDA), JA‐isoleucine (JA‐Ile), abscisic acid (ABA), and salicylic acid (SA) were extracted from 80‐mg frozen maize leaf powder in ethyl acetate spiked with isotopically labelled standards (1 ng for d_5_‐JA, d_6_‐ABA, d_6_‐SA, and ^13^C_6_‐JA‐Ile) and analysed by UHPLC–MS–MS as previously described (Glauser, Vallat, & Balmer, 2014).

### 2.7 Benzoxazinoid analysis

To evaluate the influence of volatile exposure on benzoxazinoid defence metabolites, maize leaves were measured 5 hr after simulated herbivory (*n* = 5). Seventy milligrams of frozen maize leaf powder was extracted in 700 μl of acidified H_2_O/MeOH (50:50 *v*/v; 0.1% formic acid) and then analysed with an Acquity UHPLC–MS system equipped with an electrospray source (Waters i‐Class UHPLC‐QDA, USA) using a previously established method (Robert et al., 2017). Compounds were separated on an Acquity BEH C18 column (2.1 × 100 mm i.d., 1.7‐μm particle size). Water (0.1% formic acid) and acetonitrile (0.1% formic acid) were employed as mobile phases A and B. The elution profile was 0–9.65 min, 97–83.6% A in B; 9.65–13 min, 100% B; 13.1–15 min 97% A in B. The mobile phase flow rate was 0.4 ml/min. The column temperature was maintained at 40°C, and the injection volume was 5 μl; 2‐(2‐hydroxy‐4,7‐dimethoxy‐1,4‐benzoxazin‐3‐one)‐β‐d‐glucopyranose (HDMBOA‐Glc), 2‐(2,4‐dihydroxy‐7‐methoxy‐1,4‐benzoxazin‐3‐one)‐β‐d‐glucopyranose (DIMBOA‐Glc), and 2,4‐dihydroxy‐7‐methoxy‐1,4‐benzoxazin‐3‐one (DIMBOA) were quantified in positive mode using single ion monitoring (SIM) at *m/z* 194 with cone voltage of 20 V; 2‐(2,4‐dihydroxy‐6,7‐dimethoxy‐l,4‐benzoxazin‐3‐one)‐β‐d‐glucopyranose (DIM_2_BOA‐Glc), 2‐(2,4‐dihydroxy‐1,4‐benzoxazin‐3‐one)‐β‐d‐glucopyranose (DIBOA‐Glc), 2‐(2‐hydroxy‐4,7,8‐trimethoxy‐1,4‐benzoxazin‐3‐one)‐β‐d‐glucopyranose (HDM_2_BOA‐Glc), and 6‐methoxy‐benzoxazolin‐2‐one (MBOA) were acquired in negative scan mode (*m/z* 150–650) using a cone voltage of 10 V. The ESI capillary voltage was set to 0.8 kV. The probe temperature was maintained at 600°C. The detector gain was set to 1 and the sampling frequency was 5 Hz. Absolute quantities were determined using standard curves obtained from purified or synthetic DIMBOA, DIMBOA‐Glc, HDMBOA‐Glc, and MBOA as described (Maag et al., 2015).

### 2.8 Volatile analyses

To assess the impact of volatile exposure to herbivore‐induced volatile production, maize leaves were analysed 5 hr upon simulated herbivory. At this time point, volatile priming significantly increases terpene release in maize (Engelberth et al., 2004; Erb et al., 2015). Frozen leaf powder was analysed with solid‐phase microextraction‐gas chromatography–mass spectrometry (SPME‐GC–MS; *n* = 5). This approach allows for the measurement of leaf volatile contents, which are highly correlated with volatile release rates in maize during daytime (Seidl‐Adams et al., 2015). Fifty milligrams of leaf powder were placed in a 10‐ml glass vial. An SPME fibre (100‐μm polydimethylsiloxane coating; Supelco, USA) was then inserted into the vial and incubated at 60°C for 35 min. The incubated fibre was immediately analysed by GC–MS (Agilent 7820A GC interfaced with an Agilent 5977E MSD, USA) following previously established protocols (Huang et al., 2016). Major volatile compounds were identified by comparing mass spectra with the NIST Mass Spectral Library (USA) as well as authentic standards, and the abundance of each compound was determined by integrating individual peak areas.

### 2.9 Herbivore resistance assays

To quantify the impact of volatile exposure on herbivore growth and plant resistance, individual preweighed second instar S. littoralis larvae were introduced into cylindrical mesh cages (1‐cm height and 5‐cm diameter) and then clipped onto the leaves of individual maize plants that were previously exposed to different volatile combinations (*n* = 10). The position of the cages was moved every day to provide sufficient food supply for the larvae. Larval weight was recorded 4 days after the start of the experiment. For damage quantification, the remaining leaves were scanned, and the removed leaf area was quantified with Digimizer 4.6.1 (Digimizer).

### 2.10 Statistical analyses

Gene expression, phytohormone, benzoxazinoid, volatile, larval growth, and leaf damage data were analysed by analysis of variance (ANOVA) followed by pairwise or multiple comparisons of least squares means (LSMeans), which were corrected using the false discovery rate (FDR) method (Benjamini & Hochberg, 1995). Normality was verified by inspecting residuals, and homogeneity of variance was tested through Shapiro–Wilk's tests using the “plotresid” function of the R package “RVAideMemoire” (Herve, 2015). Datasets that did not fit assumptions were log_e_‐transformed to meet the requirements of equal variance and normality. Potential synergism was evaluated using a previously described approach (Machado et al., 2018). Briefly, we calculated additive effects by randomly pairing replicates of individual volatile treatments (an indole treated plant [I_n_] and a HAC treated plant [H_n_]). For each random pair, we calculated theoretical additive values (A_n_) for the different defence parameters using the following formula: A_n_ = I_n_ + H_n_ − C_av_, where C_av_ corresponds to the average level of nonexposed control plants. The calculated additive values were then compared with the measured treatment values of the double volatile treatment using Student's *t* tests. Cases in which the measured level of the double volatile treatment was significantly greater than the calculated additive level were classified as synergistic. Principal component analysis (PCA) was furthermore employed to compare the response profiles at 0 min (defence and signalling gene expression), 45 min (signalling gene expression, phytohormones), and 5 hr (defence gene expression, benzoxazinoids, volatiles) in an integrated manner (Chapman, Schenk, Kazan, & Manners, 2002). Raw data were scaled with the “scale” function in R, and PCAs were then performed using the “MVA” function of the “RVAideMemoire” package and the “rda” function of the “vegan” package (Herve, 2015; Oksanen et al., 2013). Permutational ANOVAs were then conducted using the “adonis” function of the “vegan” package with 999 permutations. All statistical analyses were conducted with R 3.2.2 (R Foundation for Statistical Computing, Vienna, Austria) using the packages “car,” “lsmeans,” “vegan,” and “RVAideMemoire” (Bates, Machler, Bolker, & Walker, 2015; Herve, 2015; Lenth, 2016; Oksanen et al., 2013).

### 2.11 Accession numbers and data availability

The sequence data of maize genes can be found in the GenBank/EMBL database under the following accession numbers: *ZmActin* (MZEACT1G), *ZmLOX10* (DQ335768), *ZmAOS* (AY488135), *ZmPR1* (U82200), *ZmPR5* (U82201), *ZmMPI* (X78988), *ZmSerPIN* (BM382058), *ZmCyst* (CK371502), *ZmRIP2* (L26305), *ZmCYP92C5* (ACG28049), *ZmTPS2* (AY928081), *ZmTPS3* (AY928082), *ZmTPS10* (AY928078), *ZmIGL* (AF271383), *ZmBx10* (GRMZM2G311036), *ZmBx11* (GRMZM2G336824), and *ZmBx14* (GRMZM2G127418). All relevant data supporting the findings of this study can be downloaded from the Dryad repository (doi:10.5061/dryad.f21g54g).

## 3 RESULTS

### 3.1 Pre‐exposure to HAC and indole synergistically increases JA and ABA biosynthesis in induced plants

**Figure 1:**
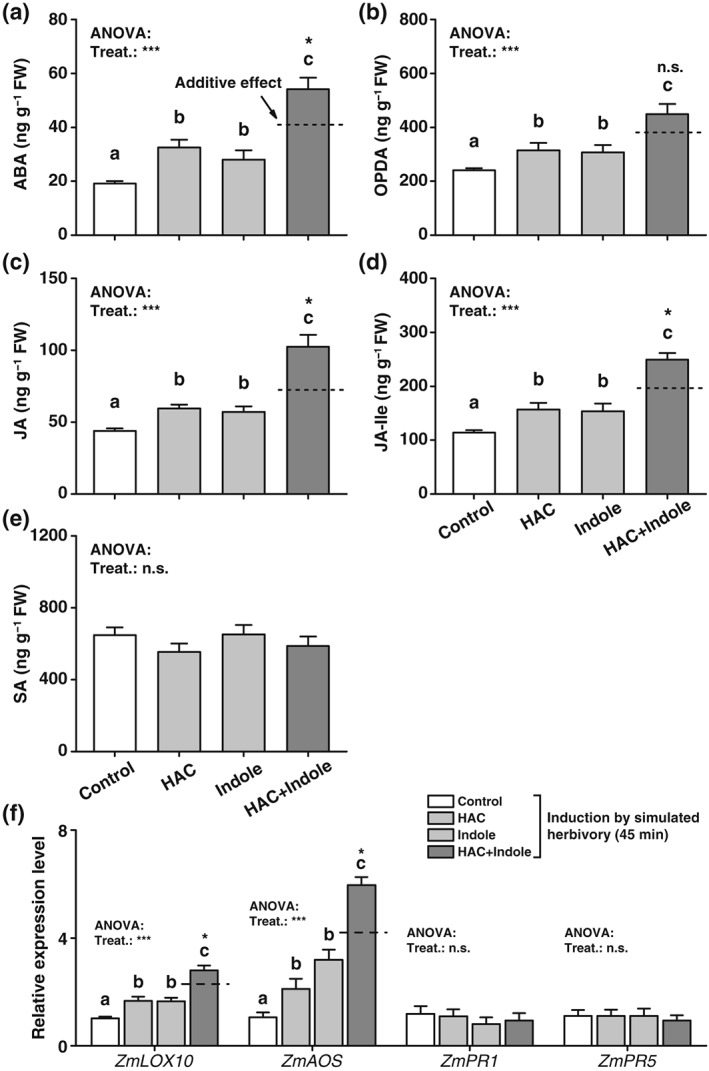
Simultaneous pre‐exposure to (*Z*)‐3‐hexenyl acetate (HAC) and indole synergistically increases abscisic acid (ABA) and jasmonic acid (JA) biosynthesis in induced maize plants. (a)‐(e) Average concentrations of the stress hormones ABA (a), 12‐oxophytodienoic acid (OPDA, b), JA (c), JA‐isoleucine (JA‐Ile, d), and salicylic acid (SA, e) in plants that were pre‐exposed to HAC, indole, or both volatiles simultaneously (HAC + Indole) and induced by simulated herbivory (+SE, *n* = 5). (f) Average transcript levels of *ZmLOX10*, *ZmAOS*, *ZmPR1*, and *ZmPR5* (+SE, *n* = 5). FW, fresh weight. n.s., not significant. Treat., treatment. Gene expression is shown relative to the expression level of the control treatment. *P* values of one‐way analyses of variance (ANOVAs) are shown (*P < 0.05, **P < 0.01, ***P < 0.001). Dashed lines indicate calculated additive effects of single volatile exposures. Letters indicate significant differences between different volatile exposure treatments (*P* < 0.05, one‐way ANOVA followed by multiple comparisons through FDR‐corrected LSMeans). Stars indicate a significant difference between the double exposure treatment and the calculated additive effect of both single treatments (*P < 0.05, Student's *t* tests)

As reported before (Engelberth et al., 2004; Erb et al., 2015), exposure to HAC and indole individually increased the production of jasmonates, including OPDA, JA, and JA‐Ile as well as ABA 45 min after induction by simulated herbivory. Simultaneous exposure to HAC and indole increased jasmonate and ABA levels beyond their calculated additive levels (Figure 1a‐d). SA levels were not changed by volatile exposure (Figure 1e). Similar to jasmonates themselves, transcript levels of the JA related genes *ZmLOX10* and *ZmAOS* (Christensen et al., 2013; Engelberth, Seidl‐Adams, Schultz, & Tumlinson, 2007) were enhanced by exposure to HAC and indole individually and synergistically increased by simultaneous HAC and indole exposure (Figure 1f). The expression levels of *ZmPR1* and *ZmPR5* were not changed by volatile exposure (Figure 1f; Morris et al., 1998). Thus, HAC and indole enhance ABA and JA biosynthesis in induced plants in a synergistic manner.

### 3.2 Pre‐exposure to HAC and indole specifically and synergistically increases the expression of defence genes in induced plants

**Figure 2:**
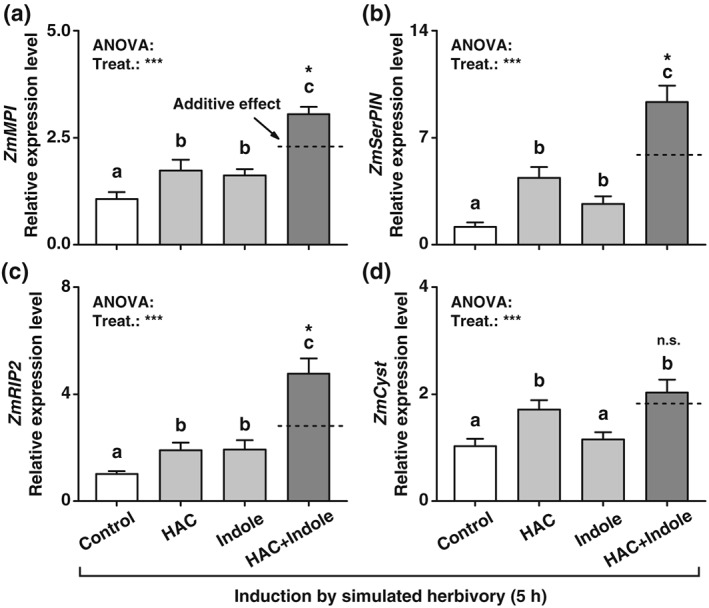
Simultaneous pre‐exposure to (*Z*)‐3‐hexenyl acetate (HAC) and indole specifically and synergistically increases defence gene expression in induced maize plants. Average transcript levels of *ZmMPI* (a), *ZmSerPIN* (b), *ZmRIP2* (c), and *ZmCyst* (d) in plants that were pre‐exposed to HAC, indole, or both volatiles simultaneously (HAC + Indole) and induced by simulated herbivory (+SE, *n* = 5). n.s., not significant. Treat., treatment. Gene expression is shown relative to the expression level of the control treatment. *P* values of one‐way analyses of variance (ANOVAs) are shown (*P < 0.05, **P < 0.01, ***P < 0.001). Dashed lines indicate calculated additive effects of single volatile exposures. Letters indicate significant differences between different volatile exposure treatments (*P* < 0.05, one‐way ANOVA followed by multiple comparisons through FDR‐corrected LSMeans). Stars indicate a significant difference between the double exposure treatment and the calculated additive effect of both single treatments (*P < 0.05, Student's *t* tests)

To further explore the interactions of HAC and indole in regulating plant defence responses, we measured the expression levels of four defensive marker genes in volatile pre‐exposed plants 5 hr after induction by simulated herbivory: the putative proteinase inhibitors *ZmMPI* (Farag et al., 2005; Tamayo, Rufat, Bravo, & San Segundo, 2000), *ZmSerPIN* and *ZmCyst* (Erb et al., 2011; Ton et al., 2007), and the insecticidal ribosome‐inactivating protein *ZmRIP2* (Chuang et al., 2014). Exposure to HAC and indole individually increased the expression of *ZmMPI*, *ZmSerPIN* , and *ZmRIP2* (Figure 2a‐c)*. ZmCyst* expression was increased by HAC, but not by indole (Figure 2d). Simultaneous exposure to HAC and indole increased the expression of *ZmMPI*, *ZmSerPIN*, and *ZmRIP2* in a synergistic manner (Figure 2a‐c). By contrast, *ZmCyst* expression was not further increased by the double volatile treatment in comparison with individual HAC exposure (Figure 2d). Thus, HAC and indole differentially regulate the expression of defence marker genes in induced plants, with combined effects ranging from neutral to synergistic.

### 3.3 Pre‐exposure to HAC and indole synergistically regulates BX biosynthesis in induced plants

**Figure 3:**
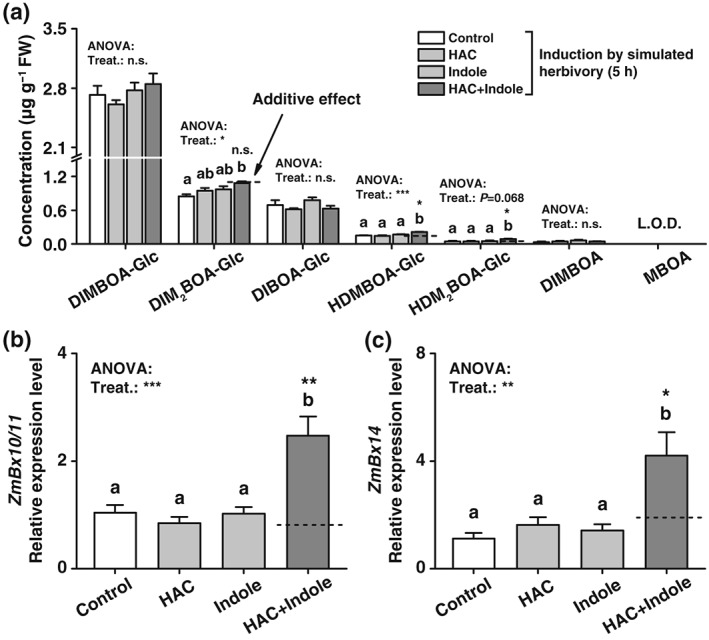
Simultaneous pre‐exposure to (*Z*)‐3‐hexenyl acetate (HAC) and indole synergistically regulates benzoxazinoid (BX) biosynthesis in induced maize plants. (a) Average concentrations of benzoxazinoids in plants that were pre‐exposed to HAC, indole, or both volatiles simultaneously (HAC + Indole) and induced by simulated herbivory (+SE, *n* = 5). (b)‐(c) Average transcript levels of *ZmBx10*/*11* and *ZmBx14* (+SE, *n* = 5). FW, fresh weight. L.O.D, below limit of detection. n.s., not significant. Treat., treatment. Gene expression is shown relative to the expression level of the control treatment. *P* values of one‐way analyses of variance (ANOVAs) are shown (*P < 0.05, **P < 0.01, ***P < 0.001). Dashed lines indicate calculated additive effects of single volatile exposures. Letters indicate significant differences between different volatile exposure treatments (*P* < 0.05, one‐way ANOVA followed by multiple comparisons through FDR‐corrected LSMeans). Stars indicate a significant difference between the double exposure treatment and the calculated additive effect of both single treatments (*P < 0.05, **P < 0.01, Student's *t* tests). DIMBOA‐Glc, 2‐(2,4‐dihydroxy‐7‐methoxy‐1,4‐benzoxazin‐3‐one)‐β‐d‐glucopyranose; DIM_2_BOA‐Glc, 2‐(2,4‐dihydroxy‐6,7‐dimethoxy‐l,4‐benzoxazin‐3‐one)‐β‐d‐glucopyranose; DIBOA‐Glc, 2‐(2,4‐dihydroxy‐1,4‐benzoxazin‐3‐one)‐β‐d‐glucopyranose; HDMBOA‐Glc, 2‐(2‐hydroxy‐4,7‐dimethoxy‐1,4‐benzoxazin‐3‐one)‐β‐d‐glucopyranose; HDM_2_BOA‐Glc, 2‐(2‐hydroxy‐4,7,8‐trimethoxy‐1,4‐benzoxazin‐3‐one)‐β‐d‐glucopyranose; DIMBOA: 2,4‐dihydroxy‐7‐methoxy‐1,4‐benzoxazin‐3‐one; MBOA: 6‐methoxy‐benzoxazolin‐2‐one

Benzoxazinoids (BXs) are important secondary metabolites, which strongly respond to herbivore attack (Glauser et al., 2011) and protect cereals against herbivores (Wouters, Blanchette, Gershenzon, & Vassao, 2016). Five hours after induction by simulated herbivory, pre‐exposure to HAC and indole individually did not significantly change the production of BXs. By contrast, simultaneous exposure to HAC and indole increased the production of HDMBOA‐Glc, DIM_2_BOA‐Glc, and HDM_2_BOA‐Glc compared with nonexposed plants (Figure 3a). HDMBOA‐Glc and HDM_2_BOA‐Glc were regulated synergistically by the two volatiles, whereas the effect on DIM_2_BOA‐Glc was not significantly different from the calculated additive effect. The expression levels of the *O*‐methyltransferases that produce HDMBOA‐Glc (*ZmBx10*/*11*, (Meihls et al., 2013)) and HDM_2_BOA‐Glc (*ZmBx14*; Handrick et al., 2016) followed the same pattern: The expression of both genes was not further increased by individual HAC or indole exposure in induced plants but strongly responded to simultaneous HAC and indole exposure (Figure 3b,c). Therefore, HAC and indole synergistically regulate the production of BXs in induced plants.

### 3.4 Pre‐exposure to HAC and indole does not synergistically regulate volatile production in induced plants

**Figure 4:**
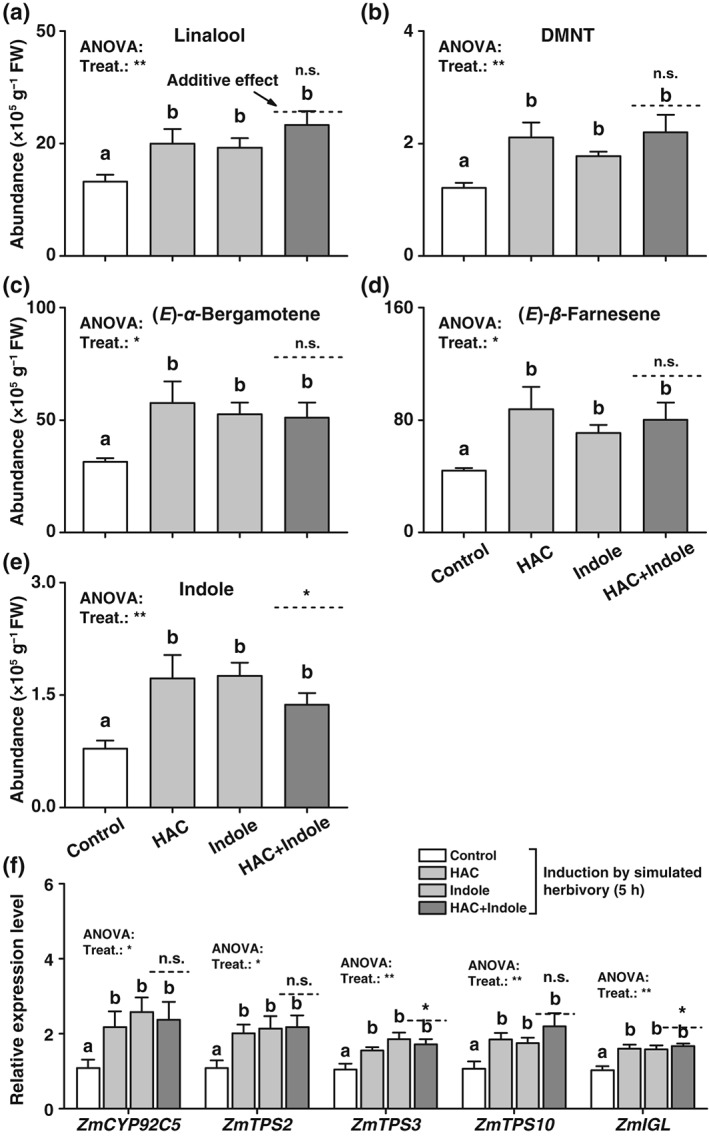
Simultaneous pre‐exposure to (*Z*)‐3‐hexenyl acetate (HAC) and indole does not synergistically regulate volatile production in induced maize plants. (a)‐(e) Average relative amounts (peak areas) of linalool (a), (3*E*)‐4,8‐dimethyl‐1,3,7‐nonatriene (DMNT, b), (*E*)‐*α*‐bergamotene (c), (*E*)‐*α*‐farnesene (d), and indole (e) in plants that were pre‐exposed to HAC, indole, or both volatiles simultaneously (HAC + Indole) and induced by simulated herbivory (+SE, *n* = 5). (f) Average transcript levels of *ZmCYP92C5*, *ZmTPS2*, *ZmTPS3*, *ZmTPS10*, and *ZmIGL* (+SE, *n* = 5). FW, fresh weight. n.s., not significant. Treat., treatment. Gene expression is shown relative to the expression level of the control treatment. *P* values of one‐way analyses of variance (ANOVAs) are shown (*P < 0.05, **P < 0.01, ***P < 0.001). Dashed lines indicate calculated additive effects of single volatile exposures. Letters indicate significant differences between different volatile exposure treatments (*P* < 0.05, one‐way ANOVA followed by multiple comparisons through FDR‐corrected LSMeans). Stars indicate a significant difference between the double exposure treatment and the calculated additive effect of both single treatments (*P < 0.05, Student's *t* tests)

Exposure of plants to both HAC and indole individually can prime herbivore‐induced terpene emissions (Engelberth et al., 2004; Erb et al., 2015). As terpene biosynthesis in maize are regulated by jasmonates (Schmelz, Alborn, Banchio, & Tumlinson, 2003; Schmelz, Alborn, & Tumlinson, 2003), we expected additive or synergistic effects of simultaneous HAC and indole exposure on volatile production similar to the defence marker genes and BXs. Exposure of maize plants to HAC and indole individually followed by simulated herbivory increased the production of linalool, (3*E*)‐4,8‐dimethyl‐1,3,7‐nonatriene (DMNT), (*E*)‐*α*‐bergamotene, (*E*)‐*α*‐farnesene and indole 5 hr after induction (Figure 4a‐e). Simultaneous exposure to HAC and indole did not further increase volatile production. For indole, we even detected significantly lower amounts in plants exposed to both volatiles than would be expected in an additive scenario. Transcript levels of genes involved in terpene synthesis, including *ZmCYP92C5*, *ZmTPS2*, *ZmTPS3*, *ZmTPS10*, and *ZmIGL* (Frey et al., 2000; Richter et al., 2016; Schnee et al., 2006), showed a similar pattern (Figure 4f).

### 3.5 Pre‐exposure to HAC and indole increases herbivore resistance of maize in an additive manner

**Figure 5:**
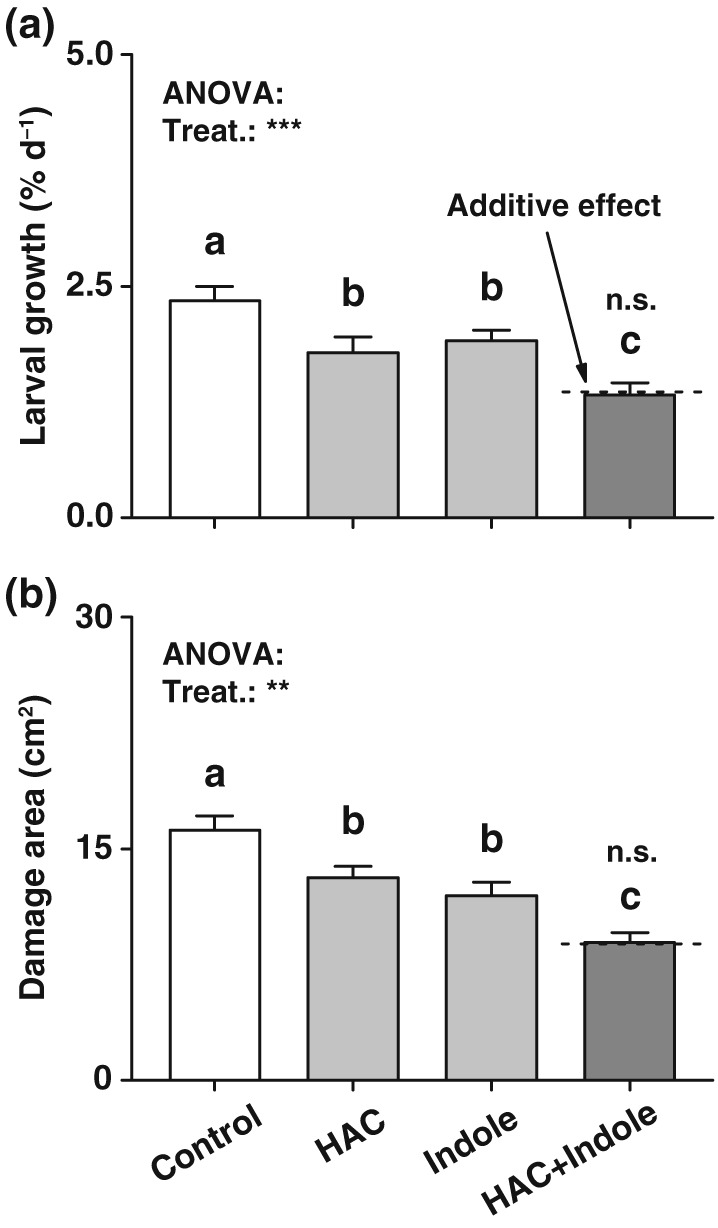
Simultaneous pre‐exposure to (*Z*)‐3‐hexenyl acetate (HAC) and indole increases herbivore resistance of maize plants. (a) Average growth rate of *Spodotera littoralis* caterpillars feeding on plants that were pre‐exposed to HAC, indole, or both volatiles simultaneously (HAC + Indole, +SE, *n* = 10). (b) Average consumed leaf area (+SE, *n* = 10). n.s., not significant. Treat., treatment. The results of one‐way analyses of variance (ANOVAs) are shown (**P < 0.01, ***P < 0.001). Dashed lines indicate calculated additive effects of single volatile exposures. Letters indicate significant differences between different volatile exposure treatments (*P* < 0.05, one‐way ANOVA followed by multiple comparisons through FDR‐corrected LSMeans)

To investigate how HAC and indole pre‐exposure influences herbivore performance and plant resistance, we measured S. littoralis growth and damage on volatile‐exposed plants. Pre‐exposure to HAC or indole individually reduced S. littoralis growth and plant damage (Figure 5). Simultaneous pre‐exposure to HAC and indole further increased this effect, with reductions of larval growth and damage attaining 40% (Figure 5). Thus, HAC and indole enhance plant resistance against herbivores in an additive manner.

### 3.6 Pre‐exposure of HAC, but not indole, directly induces defence gene expression

**Figure 6:**
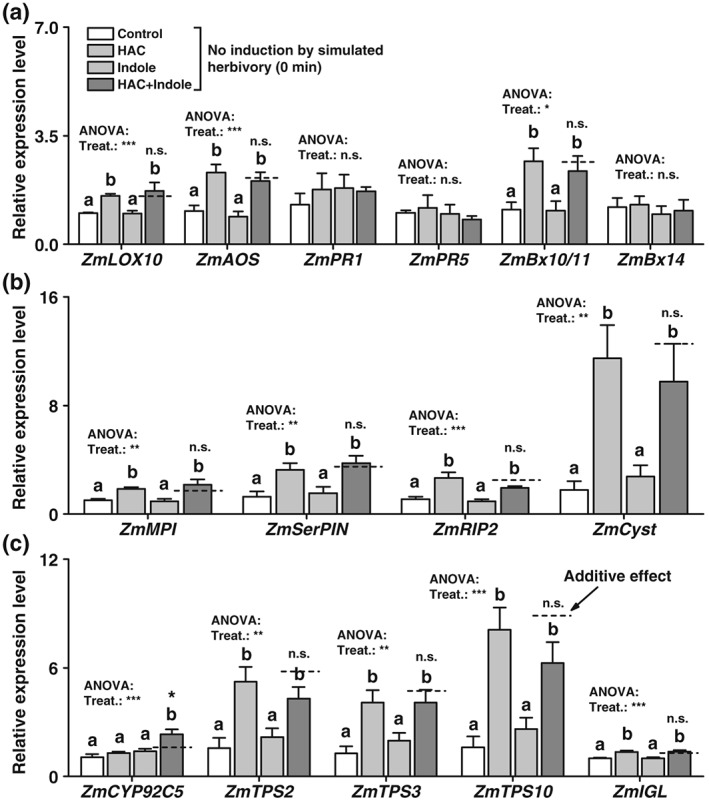
Pre‐exposure to (*Z*)‐3‐hexenyl acetate (HAC), but not indole, directly induces defence gene expression in maize plants. (a) Average transcript levels of genes involved in JA biosynthesis, SA signalling and benzoxazinoid biosynthesis in plants that were pre‐exposed to HAC, indole, or both volatiles simultaneously (HAC + Indole) without subsequent induction (+SE, *n* = 5). (b) Average transcript levels of putative proteinase inhibitors and a ribosome‐inactivating gene *ZmRIP2* (+SE, *n* = 5). (c) Average transcript levels of genes involved in terpene and indole biosynthesis (+SE, *n* = 5). n.s., not significant. Treat., treatment. Gene expression is shown relative to the expression level of the control treatment. *P* values of one‐way analyses of variance (ANOVAs) are shown (*P < 0.05, **P < 0.01, ***P < 0.001). Dashed lines indicate calculated additive effects of single volatile exposures. Letters indicate significant differences between different volatile exposure treatments (*P* < 0.05, one‐way ANOVA followed by multiple comparisons through FDR‐corrected LSMeans). Stars indicate a significant difference between the double exposure treatment and the calculated additive effect of both single treatments (*P < 0.05, Student's *t* tests)

To investigate whether the observed synergistic effects on plant defenses are due to priming or direct induction by volatile exposure, we measured the expression of the different defence marker genes upon HAC and indole exposure without further induction. HAC pre‐exposure significantly increased the expression of the tested jasmonate, volatile, and benzoxazinoid biosynthesis genes as well as other defence genes (Figure 6). By contrast, indole pre‐exposure did not directly induce any defence marker genes (Figure 6). Expression of the SA‐responsive genes *ZmPR1* and *ZmPR5* was not changed by HAC or indole exposure (Figure 6). Simultaneous exposure to HAC and indole resulted in similar gene expression patterns as HAC alone, with the exception of the DMNT biosynthesis gene *ZmCYP92C5*, whose expression was synergistically enhanced by double exposure (Figure 6). Thus, HAC, but not indole, directly induces a broad spectrum of defence genes. Furthermore, most of the synergistic effects observed upon double exposure after induction by simulated herbivory (Figures 1, 2, 3, 4) are likely due to priming rather than direct induction by HAC and indole.

### 3.7 Individual and simultaneous exposure to HAC and indole results in specific defence signatures

**Figure 7:**
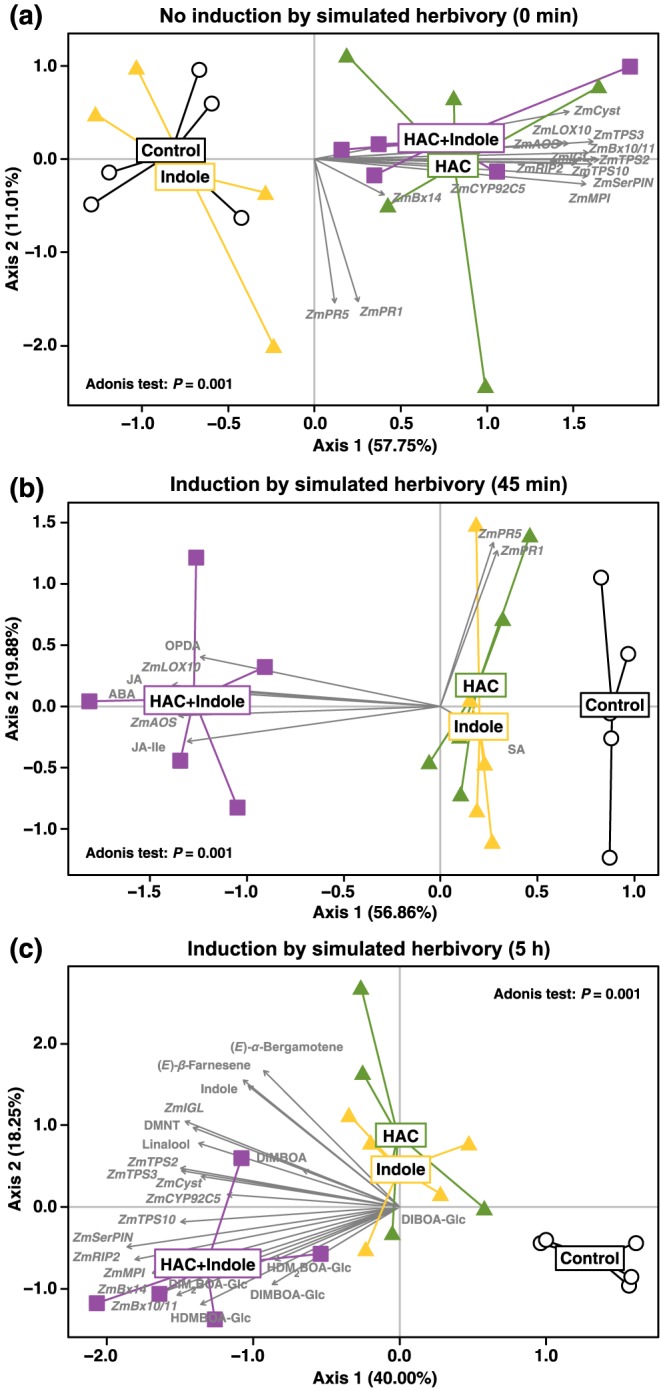
Simultaneous pre‐exposure to (*Z*)‐3‐hexenyl acetate (HAC) and indole results in specific defence signatures in maize plants. Principal component analyses of maize defence markers (a) 0 min, (b) 45 min, and (c) 5 hr after induction by simulated herbivory. Plants were pre‐exposed to HAC, indole, or both volatiles simultaneously (HAC + Indole, *n* = 5) prior to induction by simulated herbivory. PCAs include data on defence gene expression at 0 min, phytohormones and signalling related gene expression at 45 min, and defence gene expression and secondary metabolite production at the 5 hr time point. Data points represent individual replicate samples. Vectors of individual defence markers are shown as grey arrows. *P* values of permutational analyses of variance (“Adonis test”) between treatments are shown

To evaluate whether HAC and indole double exposure results in specific defence signatures, we performed PCAs for the individual time points. Permutational multivariate analysis revealed clear treatment effects at all time points (Figure 7). Without induction by simulated herbivory, HAC pre‐exposure resulted in a defence signature that was clearly separated from control and indole pre‐exposure (Figure 7a). Double‐exposure clustered together with HAC pre‐exposure (Figure 7a), reflecting the fact that indole pretreatment does not affect HAC‐induced signature changes. By contrast, 45 min and 5 hr after induction by simulated herbivory, a clear separation between controls, individual volatile exposures, and double volatile exposure was observed (Figure 7b,c). At 45 min, the treatments were predominantly separated along PC axis 1 (Figure 7b). The major vectors contributing to treatment separation were related to jasmonate and abscisic acid biosynthesis. No clear separation was observed between individual HAC and indole exposure, but double exposure was clearly separated from single exposure. The profiles at 5 hr showed a similar structure, with both PC axes 1 and 2 contributing to the separation of individual volatile exposures and double exposure (Figure 7c). In this case, the vectors contributing most to the separation of double and single exposure were benzoxazinoids and a subset of defence marker genes. Thus, double exposure to HAC and indole leads to distinct defence signatures.

## 4 DISCUSSION

Plants can perceive various environmental cues, but whether they can integrate multiple cues to regulate defence responses is poorly understood. The present study shows that simultaneous exposure of maize plants to two different herbivore‐induced volatile cues results in specific defence signatures, with most defence markers responding in an additive or synergistic fashion to double exposure. Below, we discuss the underlying mechanisms and ecological context of this phenomenon.

Maize plants that are induced by simulated herbivory responded to simultaneous HAC and indole exposure by markedly increasing their defence responses compared with nonexposed and single volatile‐exposed plants. In particular, HAC and indole synergistically enhanced the deployment of jasmonates, the expression of defence marker genes, and the production of defensive secondary metabolites in plants. Dual exposure also markedly suppressed herbivore growth and plant damage. These patterns are unlikely due to direct induction, as HAC, but not indole, directly increased defence gene expression. Instead, HAC and indole primed maize plants together to respond more strongly upon induction. A likely mechanism to explain this pattern is convergence of HAC and indole in early defence signalling. Both HAC and indole act upstream of the jasmonate signalling pathway, possibly by priming the activity of MAP kinases (Ye, Glauser, Lou, Erb, & Hu, 2018) and/or WRKY transcription factors (Engelberth, Contreras, Dalvi, Li, & Engelberth, 2013; Mirabella et al., 2015). As most of the measured downstream defenses are under the control of jasmonates (Dafoe et al., 2011; Moraes et al., 2008; Schmelz, Alborn, Banchio, et al., 2003; Stotz et al., 2002; Ton et al., 2007), the synergistic effects of HAC and indole on jasmonate signalling likely explain the enhanced defence responses observed in this study. We thus propose that maize plants can integrate two different volatile cues into early defence signalling, resulting in the amplification of a central phytohormonal signalling pathway and downstream defenses. This form of signal convergence allows for the translation of two volatile cues into a single quantitative signal, which allows plants to control the amplitude of defence and resistance expression according to the presence of different volatiles.

Apart from the amplification of jasmonate‐dependent defenses, which is similar in HAC and indole treated plants, we also observed specificity in the responses elicited by HAC and indole. For instance, HAC, but not indole, directly enhanced defence gene expression. Furthermore, although the expression of most defenses was similarly enhanced in HAC and indole‐exposed plants after elicitation, the expression of the putative proteinase inhibitor *ZmCyst* in plants induced by simulated herbivory was only enhanced in HAC exposed plants. Thus, HAC and indole differ in their effects on plant defence induction and priming and are thus likely to act via different early signalling mechanisms. We also found that simultaneous exposure to HAC and indole results in specific defence expression patterns, including synergistic effects on the production of jasmonates, defence marker genes and benzoxazinoid accumulation, and antagonistic effects on the production of volatiles such as indole in plants induced by simulated herbivory (Figure 4). Thus, the integration of two volatile cues can result in specific defence priming responses that cannot be predicted from single exposure responses and cannot be explained by signal convergence and amplification alone. In Arabidopsis thaliana, the GLV (*E*)‐2‐hexenal regulates GABA signalling and the redox status of mitochondria (Ameye et al., 2017; Mirabella et al., 2015; Scala et al., 2017). Indole on the other hand has been shown to inhibit auxin signalling in A. thaliana roots at high doses (Bailly et al., 2014). Thus, it is well possible that HAC and indole fine‐tune defence expression through signalling crosstalk (Machado et al., 2016; Pieterse, Leon‐Reyes, Van der Ent, & Van Wees, 2009), leading to specific patterns of defence priming. Further experiments aiming at understanding the early signalling events that are directly elicited by indole and HAC and how they affect hormonal signalling networks from a more holistic perspective may help to test this hypothesis.

From an ecological point of view, the integration of HAC and indole into stronger defence priming may allow plants to adjust their defence investment according to the reliability of the perceived cues. As GLVs can be emitted in response to many stresses, including for instance mechanical injury in the absence of herbivory (Ebel et al., 1995; Scala et al., 2013), they cannot be used as reliable cues by plants to anticipate herbivory. The same is true for indole alone, which can emanate from various environmental sources (Bailly et al., 2014; Stamm et al., 2005) but is emitted from leaves in much greater quantities upon contact with herbivore‐elicitors than wounding alone (Frey et al., 2000; Zhuang et al., 2012). The simultaneous presence of indole and GLVs on the other hand may be a relatively robust predictor of herbivore attack due to the complementary nature of their information contents. Given that priming can be costly (van Hulten, Pelser, van Loon, Pieterse, & Ton, 2006), adjusting the magnitude of priming according to the reliability of the perceived cues may be beneficial. Especially when the reliability of individual volatile cues is low, the ability to integrate multiple volatile cues may confer important advantages to plants. However, it is important to point out that the integration of multiple volatile cues is not always necessary to obtain reliable information from the environment. Insect pheromones, for instance, can be fairly specific and may be sufficient to reliably indicate the presence of a herbivore. In line with this argument, Solidago altissima plants respond similarly to the exposure to a single pheromone component of the goldenrod gall fly as to the full volatile blend of the herbivore (Helms et al., 2017).

Double exposure to HAC and indole enhanced direct defenses but had no clear effect on the emission of induced volatiles, which are often viewed as indirect defenses that attract natural enemies (Turlings & Erb, 2018). Recent work in tomato furthermore demonstrates that changes in light quality leading to phytochrome B inactivation shifts tomato defenses from direct to volatile‐mediated indirect defenses (Cortés, Weldegergis, Boccalandro, Dicke, & Ballaré, 2016). Thus, plants seem to be able to integrate various environmental cues to regulate their relative investment into direct and indirect defenses. Regarding the results of the present study however, we would like to remain cautious with our interpretation, as the effects of the observed patterns on indirect defenses have not been quantified, and the ecological interpretation of defence responses of a domesticated plant warrants caution due to possible pleiotropic effects of domestication. Nevertheless, exploring if and how the composition of volatile cues influences the relative investment of plants into direct and indirect defenses is an exciting prospect of this work.

## 5 CONCLUSIONS

Plants perceive a variety of volatiles from the environment. Our work lends support to the concept that plants are also able to integrate multiple volatile cues into specific, and possibly adaptive, defence responses. Understanding the mechanisms and ecological factors that shape the evolution of signal integration will be important to improve our understanding of plant responses to complex volatile blends.

## AUTHOR CONTRIBUTIONS

L. H. conceived, designed, performed, and analysed experiments and wrote the first draft of the manuscript. M. Y. designed, performed, and analysed experiments. M. E. acquired funding, conceived the project, designed, supervised, and analysed experiments, and wrote the first draft of the manuscript. All authors contributed to the final version of the paper.

## Supporting information


**Table S1**. Primers used for quantitative real time PCR of target genes
