# Supplementary material for: Integration of two herbivore‐induced plant volatiles results in synergistic effects on plant defence and resistance
Source: Plant Cell Environ. 2018 Oct 16;42(3):959–71. doi: 10.1111/pce.13443 (PMC6392123; doi:10.1111/pce.13443)
Supplement: Supplementary file 1 — Table S1. Primers used for quantitative real time PCR of target genes [file PCE-42-959-s001.docx]

## Supporting Information

**Article title**: Integration of two herbivore-induced plant volatiles results in synergistic effects on plant defense and resistance

**Authors**: Lingfei Hu, Meng Ye and Matthias Erb

**Table S1. Primers used for quantitative real time PCR of target genes**

| Gene name | Forward primer (5’-…-3’) | Reverse primer (5’-…-3’) | Reference |
| --- | --- | --- | --- |
| *ZmActin* | ccatgaggccacgtacaact | GGTAAAACCCCCACTGAGGA | ([Erb *et al.*, 2009](#_ENREF_3)) |
| *ZmLOX10* | ATCCTCAGCATGCATTAGTCC | AGTCTCAAACGTGCCTCTTGT | ([Christensen *et al.*, 2013](#_ENREF_1)) |
| *ZmAOS* | acctgttcacgggcacctac | CGAGGAGCGAGGAGAAGTTG | ([Erb *et al.*, 2009](#_ENREF_3)) |
| *ZmRIP2* | gagatccccgacatgaagga | CTGCGCTGCTGCGTTTT | ([Chuang *et al.*, 2014](#_ENREF_2)) |
| *ZmPR1* | ctgggtgtccgagaagcagt | CGGGTTGTAGCTGCAGATGAT | ([Morris *et al.*, 1998](#_ENREF_6)) |
| *ZmPR5* | tgcatgcatgggctagtgat | CGCACACAAATCCAGCTACG | ([Morris *et al.*, 1998](#_ENREF_6)) |
| *ZmMPI* | atgagctccacggagtgc | TCAGCCGATGTGGGGCGTC | ([Ton *et al.*, 2007](#_ENREF_9)) |
| *ZmCyst* | ggacatgagctggcgatttt | CAAGGAGCACAACAGGCAGA | ([Ton *et al.*, 2007](#_ENREF_9)) |
| *ZmSerPIN* | acctgatgcactgcttgcac | GACGGAGGAGGAAGGAGGAG | ([Ton *et al.*, 2007](#_ENREF_9)) |
| *ZmTPS2* | AAGCAGACCTGCACGAGGTT | GAAGGAGCATGGATCTAACCATG | ([Richter *et al.*, 2016](#_ENREF_7)) |
| *ZmTPS3* | AAGGAGACCTGCGCGATGTC | CAAGGAGCATGGATCTAACCCTA | ([Richter *et al.*, 2016](#_ENREF_7)) |
| *ZmTPS10* | tgtgtccacggtccaatgtt | GTCCGCTGTCCTTGCAAAAT | ([Schnee *et al.*, 2006](#_ENREF_8)) |
| *ZmCYP92C5* | ACGACCTTCACGACCATTTC | CCTCATCCAGGACATCATCG | ([Richter *et al.*, 2016](#_ENREF_7)) |
| *ZmIGL* | gcctcatagttcccgacctc | GAATCCTCGTGAAGCTCGTG | ([Frey *et al.*, 2000](#_ENREF_4)) |
| *ZmBx10/11* | CAGCAGGTGGTGGTGATAAT | AGCGCCAGACTCACAAAGG | ([Maag *et al.*, 2016](#_ENREF_5)) |
| *ZmBx14* | GAAAGCCGCTTCTTGATGCC | GGAACATATTGCCCGCAACG | ([Maag *et al.*, 2016](#_ENREF_5)) |

**References:**

Christensen S.A., Nemchenko A., Borrego E., Murray I., Sobhy I.S., Bosak L., DeBlasio S., Erb M., Robert C.A.M., Vaughn K.A., Herrfurth C., Tumlinson J., Feussner I., Jackson D., Turlings T.C.J., Engelberth J., Nansen C., Meeley R. & Kolomiets M.V. (2013) The maize lipoxygenase, *ZmLOX10*, mediates green leaf volatile, jasmonate and herbivore-induced plant volatile production for defense against insect attack. *Plant Journal*, **74**, 59-73.

Chuang W.P., Herde M., Ray S., Castano-Duque L., Howe G.A. & Luthe D.S. (2014) Caterpillar attack triggers accumulation of the toxic maize protein RIP2. *New Phytologist*, **201**, 928-939.

Erb M., Flors V., Karlen D., de Lange E., Planchamp C., D'Alessandro M., Turlings T.C.J. & Ton J. (2009) Signal signature of aboveground-induced resistance upon belowground herbivory in maize. *Plant Journal*, **59**, 292-302.

Frey M., Stettner C., Pare P.W., Schmelz E.A., Tumlinson J.H. & Gierl A. (2000) An herbivore elicitor activates the gene for indole emission in maize. *Proceedings of the National Academy of Sciences of the United States of America*, **97**, 14801-14806.

Maag D., Kohler A., Robert C.A.M., Frey M., Wolfender J.L., Turlings T.C.J., Glauser G. & Erb M. (2016) Highly localized and persistent induction of *Bx1*-dependent herbivore resistance factors in maize. *Plant Journal*, **88**, 976-991.

Morris S.W., Vernooij B., Titatarn S., Starrett M., Thomas S., Wiltse C.C., Frederiksen R.A., Bhandhufalck A., Hulbert S. & Uknes S. (1998) Induced resistance responses in maize. *Molecular Plant-Microbe Interactions*, **11**, 643-658.

Richter A., Schaff C., Zhang Z., Lipka A.E., Tian F., Kollner T.G., Schnee C., Preiss S., Irmisch S., Jander G., Boland W., Gershenzon J., Buckler E.S. & Degenhardt J. (2016) Characterization of biosynthetic pathways for the production of the volatile homoterpenes DMNT and TMTT in *Zea mays*. *Plant Cell*, **28**, 2651-2665.

Schnee C., Köllner T.G., Held M., Turlings T.C., Gershenzon J. & Degenhardt J. (2006) The products of a single maize sesquiterpene synthase form a volatile defense signal that attracts natural enemies of maize herbivores. *Proceedings of the National Academy of Sciences of the United States of America*, **103**, 1129-1134.

Ton J., D'Alessandro M., Jourdie V., Jakab G., Karlen D., Held M., Mauch-Mani B. & Turlings T.C.J. (2007) Priming by airborne signals boosts direct and indirect resistance in maize. *Plant Journal*, **49**, 16-26.
